# Examining Cesarean Delivery Rates by Race: a Population-Based Analysis Using the Robson Ten-Group Classification System

**DOI:** 10.1007/s40615-020-00842-3

**Published:** 2020-08-17

**Authors:** Elise G. Valdes

**Affiliations:** Relias LLC, Relias Institute, 1010 Sync St., Morrisville, NC 27560 USA

**Keywords:** Cesarean section, Racial disparities, TGCS

## Abstract

The Robson Ten-Group Classification System is widely considered to be the gold standard for comparing cesarean section (CS) delivery rates, despite limited adoption in the United States (US). When reporting overall CS rates, Blacks and other minorities are typically reported to have high CS rates but comparing overall CS rates may be misleading as CS may be more common in some higher risk populations. Improved understanding of how CS rates differ by race among standardized groups could highlight differences in care and areas for improvement. The current study examines racial differences in cesarean section delivery rates using the Robson Ten-Group Classification System in a nationwide sample. Data from US vital statistics live birth certificates were used to identify 3,906,088 births which were each classified into one of the ten groups based on five obstetric characteristics identifiable on presentation for delivery including parity, onset of labor, gestational age, fetal presentation, and number of fetuses. Results indicated that Black and Asian mothers had the highest CS rates in groups 1–4 which all contain single, cephalic pregnancies at term with no prior CS and are only differentiated by parity and onset of labor. Black mothers also had the lowest CS rates for groups 6 and 7, containing women with nulliparous and multiparous breech births. Black and Asian mothers show differences in CS rates among groups that could indicate lack of appropriate care. Efforts should be made to prevent unnecessary primary CS among low-risk mothers.

## Introduction

High cesarean section (CS) rates in the United States (US) and around the world have gained much attention in recent decades from obstetricians and academics due to the cost and risks associated with CS. Numerous regional- or hospital-level studies in the US have documented significantly higher rates of CS among African American women since the 1990s and Asian and Pacific Islander women in the last decade compared with other races. Racial differences in CS, however, have not been examined in a nationwide cohort in nearly 15 years, limiting generalizability [1–3]. Despite numerous local studies reporting differences in obstetric care by race, reasons for these disparities are still unclear. The purpose of the current paper is to gain generalizable insight into factors that might be driving interracial disparities in CS by applying the Robson Ten Group Classification System (TGCS) by race to a national sample.

While CS can be beneficial to mothers and babies when medically necessary, systemic overuse can lead to increasing medical costs as well as increased risk for poor maternal and fetal outcomes. In the US, African American women are significantly more likely to have a cesarean section than women of all other races, with estimates ranging from 22 to 64% increased incidence [e.g., 4–6]. Similarly, rates of CS are particularly high among Asian and Pacific Islanders in the US who are 19 to 49% more likely to have a CS compared with White women [4, 7]. Most of the current studies investigating racial differences in CS and obstetric care have been done in specific cities [8] and states [6, 9, 10] and in individual hospitals [4, 11, 12] or hospital systems [7, 13]. The last investigation of CS rates by race using nationwide data was from 2006 and contained somewhat conflicting results.

A major limitation of prior research is the focus on comparing total CS rates which can be misleading because CS is necessary when medically indicated and may be more common in some populations such as women with prior CS, multiple fetuses, or atypical fetal lies. If populations of laboring women vary, comparing total CS rates may provide an inaccurate story. Instead of comparing total CS rates, the World Health Organization (WHO) has recommended the Robson TGCS as the global standard for assessing, monitoring, and comparing CS rates [14]. The TGCS classifies all women admitted for delivery into 10 mutually exclusive and all-inclusive groups based on 5 obstetric characteristics that are routinely collected by obstetrical providers [15, 16]. The information coming from the Robson TGCS can be a useful tool to inform practice, as it stratifies women into uniform groups and allows easy comparisons with others using the system.

The Robson TGCS is widely used internationally but has not been used often within the US. The study of Hehir and colleagues [17] was the first and only study to date to apply the Robson TGCS to a nationwide cohort in the United States (US). They found that between 2005 and 2014 women with prior CS represent an increasing proportion of cesarean deliveries in the US. Despite the benefits of this first examination, further research is needed to explain the racial and ethnic differences in women who undergo cesarean sections.

A better understanding of how CS rates in the US differ by race across a range of clinical scenarios and indications using a nationwide sample could highlight differences in care and areas for further investigation. Using the TGCS will allow standardized comparisons of data and help identify the clinical scenarios behind the changes in CS rates. The goal of the current study is to compare racial differences in CS rates across the TGCS in a nationwide cohort to better understand the clinical scenarios driving differences in CS rates. We aim to answer the following questions: Are there significant differences in CS rates by race in the US? If so, what factors might contribute to those differences?

## Materials and Methods

### Study Data and Measures

Data for the current study were obtained from the US vital statistics available from the live birth certificate. The dataset is assembled, maintained, and provided by the National Vital Statistics System, a joint effort of the National Center for Health Statistics and states to provide access to statistical information from birth certificates. Completion of birth certificates is required for all births, and federal law mandates national collection and publication of birth statistics.

#### Study Cohort

The study cohort consisted of all women who delivered live births in the United States in 2016 and had complete data on the variables listed below (*n* = 3,906,088). From the original sample of 3,956,112 women, 50,024 were excluded for missing data, accounting for less than 1.2% of the population.

#### Demographics

Demographic data included age in years, education, race, primiparity, singleton birth, smoking status, pre-term delivery, and mode of delivery.

#### Race

The variable of race was coded into 6 categories: White, Black, American Indian or Alaskan Native (AIAN), Asian, Native Hawaiian or Other Pacific Islander (NHOPI), and more than one race.

#### The Robson Ten-Group Classification System

The TGCS classifies women into 10 mutually exclusive and all-inclusive groups based on 5 obstetric characteristics, including parity (nulliparous, multiparous without a uterine scar, multiparous with a uterine scar), onset of labor (spontaneous, induced, or CS before labor), gestational age (≥ 37 weeks or < 36 weeks), fetal presentation (cephalic, breech, transverse, or oblique), and number of fetuses (singleton or multiple deliveries). As these variables are routinely collected by obstetrical providers and included in the vital statistics dataset, the women in our sample are easily classifiable using the TGCS [15].

### Statistical Analysis Plan

For this retrospective cross-sectional study, we report the maternal sociodemographic characteristics and clinical and obstetric factors including maternal age, primiparity, education, race, plurality, smoking during pregnancy, and mode of delivery of the overall sample (all available in the vital statistics dataset).

Women were separated into groups based on the racial categories described above. Additionally, women were categorized into one of the ten TGCS groups via statistical syntax coding using SPSS version 26. We calculated the CS rate by race for each of the ten groups. To compare CS rates by race among the different TGCS groups, *z*-tests were used to compare column proportions, which included a Bonferroni correction for multiple comparisons. Bonferroni adjusted *p* values are presented.

## Results

Women aged 25–35 comprised over half the delivering women in 2016 with 28.6% of women completing some college. White and Black women were the largest racial groups comprising 73.6% and 15.7% of the sample, respectively. Single births were most common (96.5%) and less than 10% of women smoked at any point during their pregnancy. CS accounted for 31.8% of all births. For complete demographic characteristics, see Table 1.

**Table 1:** Demographic characteristics of all delivering women in the US in 2016

	*n* = 3,906,088	%
Maternal age		
< 20	209,789	5.4
20–24	795,200	20.4
25–29	1,137,643	29.1
30–34	1,100,550	28.2
35–39	542,049	13.9
40–44	111,877	2.9
45–49	8188	0.2
> 50	791	< 0.01
Primiparity	1,220,101	31.3
Maternal education		
< 9 years	130,173	3.3
9–11 years	401,994	10.3
12 years	968,332	24.8
13–15 years	1,117,992	28.6
16 years	780,299	20.0
18 years	356,262	9.1
20 years	101,693	2.6
Unknown	49,343	1.3
Race		
White	2,874,450	73.6
Black	614,305	15.7
AIAN	37,358	1.0
Asian	272,990	7.0
NHOPI	11,263	0.3
More than one	95,722	2.5
Singleton	3,770,012	96.5
Smoked during pregnancy (any)	279,016	7.1
Pre-term delivery (< 37 weeks)	447,621	11.5
Mode of delivery		
Vaginal	2,589,178	66.3
VBAC	74,744	1.9
Primary CS	716,644	18.3
Repeat CS	524,149	13.4
Vaginal - unknown prior CS	801	< 0.01
CS - unknown prior CS	367	< 0.01

*AIAN* American Indian or Alaskan Native, *NHOPI* Native Hawaiian or Other Pacific Islander, *VBAC* vaginal birth after cesarean section, *CS* cesarean section

Column proportion tests were conducted to compare CS rates across races for each of the TGCS groups (see Table 2). For overall CS rate, Blacks had significantly higher CS rates than every other racial group (*p*’s < .001). Asians had significantly higher CS rates than every other racial group except Blacks (*p*’s < .001). Whites had higher CS rates than more than one race and AIAN (*p*’s < .001). AIAN had significantly lower CS rates than every other racial group (*p*’s < .001, except when compared with NHOPI, *p* = .002).

**Table 2:** Cesarean section rates by race across Robson groups

Robson group	White (A)	Black (B)	AIAN (C)	Asian (D)	NHOPI (E)	More than one (F)
Overall	31.0%^C,F^ (891,133/2874450)	35.4%^A,C,D,E,F^ (217,196/614305)	28.1% (10,481/37358)	33.1%^A,C,E,F^ (90,288/272990)	29.9%^C^ (3368/11263)	30.0%^C^ (28,694/95722)
1 - Nulliparous, singleton, cephalic, ≥ 37 weeks, in spontaneous labor	11.6% (53,457/460331)	14.0%^A,C,D,F^ (11,709/83803)	10.4% (514/4921)	12.6%^A,C,F^ (7218/57324)	13.6%^C^ (220/1613)	11.7% (2002/17146)
2a - Nulliparous, singleton, cephalic, ≥ 37 weeks, induced labor	24.6% (58,344/237497)	30.2% ^A,C,D,F^ (12,174/40330)	26.1% (654/2508)	26.2%^A^ (5223/19965)	28.6% (157/549)	25.8% (2071/8025)
2b - Nulliparous, singleton, cephalic, ≥ 37 weeks, pre-labor cesarean delivery	100.0% (72,310/72310)	100.0% (16,558/16558)	100.0% (445/445)	100.0% (10,939/10939)	100.0% (220/220)	100.0% (2354/2354)
3 - Multiparous (excluding previous cesareans), single, cephalic, ≥ 37 weeks, in spontaneous labor	3.5% (28,995/828874)	5.6% ^A,C,D,E,F^ (9755/173022)	3.4% (402/11936)	3.9%^A^ (3048/79058)	3.6% (136/3758)	4.4%^A,C,D^ (1216/27696)
4a - Multiparous (excluding previous cesareans), single, cephalic, ≥ 37 weeks, induced labor	7.4% (29,142/391576)	12.2%^A,C,D,E,F^ (8744/71433)	7.1% (374/5297)	9.7%^A,C^ (2228/22944)	8.7% (94/1078)	9.3%^A,C^ (1085/11725)
4b - Multiparous (excluding previous cesareans), single, cephalic, ≥ 37 weeks, pre-labor cesarean delivery	100.0% (88,888/88888)	100.0% (23,179/23179)	100.0% (886/886)	100.0% (7907/7907)	100.0% (281/281)	100.0% (2624/2624)
5 - Previous cesarean delivery, single, cephalic, ≥ 37 weeks	86.6%^E^ (305,424/352499)	86.6%^E^ (69,367/80138)	85.4%^E^ (3779/4426)	86.6%^E^ (30,296/34996)	77.6% (1158/1492)	86.1%^E^ (9309/10810)
6 - All nulliparous breeches	95.7%^B^ (33,408/34902)	89.6% (3759/4196)	94.3% (266/282)	96.4%^B^ (3778/3918)	91.9% (79/86)	94.7%^B^ (901/951)
7 - All multiparous breeches (including previous cesareans)	93.3%^B^ (50,212/53796)	90.2% (9548/10585)	92.1% (748/812)	93.9%^B^ (4441/4731)	92.5% (271/293)	92.6%^B^ (1468/1585)
8 - All multiple deliveries (including previous cesareans)	73.6%^C^ (72,089/97993)	73.7%^C^ (18,299/24824)	65.0% (672/1034)	80.3%^A,B,C,E,F^ (6962/8668)	66.6% (197/296)	71.8%^C^ (2343/3261)
9 - All transverse and oblique lies (including previous cesareans)	59.3%^B,D,F^ (20,432/34470)	52.3% (4107/7852)	75.7%^A,B,D,E,F^ (435/575)	50.7% (1421/2805)	57.0% (114/200)	51.4% (570/1109)
10 - All pre-term, single, cephalic, ≤ 36 weeks (including previous cesareans)	35.4%^C,E,F^ (78,432/221314)	38.3%^A,C,D,E,F^ (29,997/78385)	30.8% (1306/4236)	34.6% (6827/19735)	31.6%^C,F^ (441/1397)	32.6% (2751/8436)

For each statistically significant pair (Bonferroni adjusted, *p* < .05), the key of the racial group(s) with a significantly lower CS rate appears in superscript

*AIAN* American Indian or Alaskan Native, *NHOPI* Native Hawaiian or Other Pacific Islander

For Robson group 1 (nulliparous, singleton, cephalic, ≥ 37 weeks, in spontaneous labor), Black mothers had significantly higher CS rates than Whites, AIAN, Asians, and more than one race (*p*’s < .001). Asians had higher CS rates than Whites (*p* < .001), AIAN (*p* < .001), and more than one race (*p* = .02). NHOPI had higher CS rates than AIAN (*p* = .006).

For Robson group 2.1 (nulliparous, singleton, cephalic, ≥ 37 weeks, induced labor), Blacks had significantly higher CS rates than Whites, AIAN, Asians, and more than one race (*p* < .001). Asians had higher CS rates than Whites (*p* < .001).

For Robson group 3 (multiparous, excluding previous cesareans, singleton, cephalic, ≥ 37 weeks, in spontaneous labor), Blacks had significantly higher CS rates than every other racial group (*p*’s < .001). Asians had higher CS rates than Whites (*p* < .001). More than one race had significantly higher CS rates than Whites (*p* < .001), AIAN (*p* < .001), and Asians (*p* = .001).

For Robson group 4.1 (multiparous, excluding previous cesareans, singleton, cephalic, ≥ 37 weeks, induced labor), Blacks had significantly higher CS rates than every other racial group (*p*’s < .001, except when compared with Asians, *p* = .007). Asians and more than one race had higher CS rates than Whites and AIAN (*p*’s < .001).

For Robson group 5 (previous cesarean delivery, singleton, cephalic, ≥ 37 weeks), NHOPI had significantly lower CS rates than every other race (*p*’s < .001).

For Robson group 6 (all nulliparous breeches), Whites, Asians, and more than one race had significantly higher CS rates than Blacks (*p*’s < .001).

For Robson group 7 (all multiparous breeches, including previous cesareans), Whites, Asians, and more than one race had significantly higher CS rates than Blacks (respectively: *p* < .001, *p* < .001, *p* = .04).

For Robson group 8 (all multiple deliveries, including previous cesareans), Asians had significantly higher CS rates than every other racial group (*p*’s < .001). Whites, Blacks, and more than one race had significantly higher CS rates than AIAN (*p*’s ≤ .001).

For Robson group 9 (all transverse and oblique lies, including previous cesareans), AIAN had significantly higher CS rates than every other racial group (*p*’s < .001). Whites had significantly higher CS rates than Blacks, Asians, and more than one race (*p*’s < .001).

For Robson group 10 (all pre-term, singleton, cephalic, ≤ 36 weeks, including previous cesareans), Blacks had significantly higher CS rates than every other racial group (*p*’s < .001). Whites had significantly higher CS rates than AIAN (*p* < .001), NHOPI (*p* = .04), and more than one race (*p* < .001). Asians had higher CS rates than AIAN (*p* < .001) and more than one race (*p* = .02).

For reference, Tables 3 and 4 show the contribution of each group of the Ten-Group Classification to the overall obstetric population by race and the contribution of each group of the Ten-Group Classification to the overall rate of cesarean sections by race, respectively. No statistical analyses were conducted with these data.

**Table 3:** The contribution of each group of the Ten Group Classification to the overall obstetric population by race

Robson group	White (*n* = 2,874,450)	Black (*n* = 614,305)	AIAN (*n* = 37,358)	Asian (*n* = 272,990)	NHOPI (*n* = 11,263)	More than one (*n* = 95,722)
1	16.0% (460331)	13.6% (83803)	13.2% (4921)	21.0% (57324)	14.3% (1613)	17.9% (17146)
2a	8.3% (237497)	6.6% (40330)	6.7% (2508)	7.3% (19965)	4.9% (549)	8.4% (8025)
2b	2.5% (72310)	2.7% (16558)	1.2% (445)	4.0% (10939)	2.0% (220)	2.5% (2354)
3	28.8% (828874)	28.2% (173022)	32.0% (11936)	29.0% (79058)	33.4% (3758)	28.9% (27696)
4a	13.6% (391576)	11.6% (71433)	14.2% (5297)	8.4% (22944)	9.6% (1078)	12.2% (11725)
4b	3.1% (88888)	3.8% (23179)	2.4% (886)	2.9% (7907)	2.5% (281)	2.7% (2624)
5	12.3% (352499)	13.0% (80138)	11.8% (4426)	12.8% (34996)	13.2% (1492)	11.3% (10810)
6	1.2% (34902)	0.7% (4196)	0.8% (282)	1.4% (3918)	0.8% (86)	1.0% (951)
7	1.9% (53796)	1.7% (10585)	2.2% (812)	1.7% (4731)	2.6% (293)	1.7% (1585)
8	3.4% (97993)	4.0% (24824)	2.8% (1034)	3.2% (8668)	2.6% (296)	3.4% (3261)
9	1.2% (34470)	1.3% (7852)	1.5% (575)	1.0% (2805)	1.8% (200)	1.2% (1109)
10	7.7% (221314)	12.8% (78385)	11.3% (4236)	7.2% (19735)	12.4% (1397)	8.8% (8436)

*AIAN* American Indian or Alaskan Native, *NHOPI* Native Hawaiian or Other Pacific Islander

**Table 4:** The contribution of each group of the Ten Group Classification to the overall rate of cesarean sections by race

Robson group	White (*n* = 891,133)	Black (*n* = 217,196)	AIAN (*n* = 10,481)	Asian (*n* = 90,288)	NHOPI (*n* = 3368)	More than one (*n* = 28,694)
1	6.0% (53457)	5.4% (11709)	4.9% (514)	8.0% (7218)	6.5% (220)	7.0% (2002)
2a	6.5% (58344)	5.6% (12174)	6.2% (654)	5.8% (5223)	4.7% (157)	7.2% (2071)
2b	8.1% (72310)	7.6% (16558)	4.2% (445)	12.1% (10939)	6.5% (220)	8.2% (2354)
3	3.3% (28995)	4.5% (9755)	3.8% (402)	3.4% (3048)	4.0% (136)	4.2%(1216)
4a	3.3% (29142)	4.0% (8744)	3.6% (374)	2.5% (2228)	2.8% (94)	3.8% (1085)
4b	10.0% (88888)	10.7% (23179)	8.5% (886)	8.8% (7907)	8.3% (281)	9.1% (2624)
5	34.3% (305424)	31.9% (69367)	36.1% (3779)	33.6% (30296)	34.4% (1158)	32.4% (9309)
6	3.7% (33408)	1.7% (3759)	2.5% (266)	4.2% (3778)	2.3% (79)	3.1% (901)
7	5.6% (50212)	4.4% (9548)	7.1% (748)	4.9% (4441)	8.0% (271)	5.1% (1468)
8	8.1% (72089)	8.4% (18299)	6.4% (672)	7.7% (6962)	5.8% (197)	8.2% (2343)
9	2.3% (20432)	1.9% (4107)	4.2% (435)	1.6% (1421)	3.4% (114)	2.0% (570)
10	8.8% (78432)	13.8% (29997)	12.5% (1306)	7.6% (6827)	13.1% (441)	9.6% (2751)

*AIAN* American Indian or Alaskan Native, *NHOPI* Native Hawaiian or Other Pacific Islander

## Discussion

The goal of the current study was to gain insight into factors that are potential causes of observed interracial disparities in CS on a national scale by applying the Robson Ten-Group Classification System (TGCS) by race. Our results are aligned with previous research indicating that Blacks had significantly higher rates of overall CS than all other races. Asians also had higher overall CS rates than other races, except for Blacks. Interestingly, AIAN had the lowest overall CS rates.

Analysis of the CS rates using the Robson TGCS revealed that Blacks had the highest rates of CS in Robson groups 1–4, which all contain women with a singleton, cephalic pregnancy, at term, with no prior CS, and are only differentiated by parity and onset of labor. Asian mothers also had significantly higher CS rates in these groups than White mothers. These groups are considered low-risk and are typically the most favorable for vaginal delivery [18], suggesting that the effort of providers should focus on patient safety and the prevention of primary CS among low-risk Black and Asian mothers. Among these low-risk groups, CS is typically performed for labor complications such as dystocia or fetal distress. Previous research has suggested that indications for CS may also vary by race with Black women being more likely than White women to undergo a CS for subjective indicators such as fetal distress or failure to progress [4]. Further research should explore clinical decision-making surrounding CS. The significantly higher CS rates among Blacks in Robson group 4a (containing singleton, cephalic, multiparous women at term with no prior CS) compared with those among all other racial groups could suggest suboptimal case selection and mode of induction [19]. There is a dearth of research examining differences in indications for induction by race. Research has shown that Black women are more likely than White women to undergo CS for subjective indicators [4], and future research should investigate if indications for induction also vary by race.

Robson group 5 contains women with prior CS delivery and as the rates of vaginal birth after CS (VBAC) are still relatively low in the US [17], it is unsurprising to find few disparities between the racial groups; however, it is of note that NHOPI had significantly lower CS rates by almost 10%. After an NIH recommendation in 1980 endorsing trial of labor after CS to help limit rising CS rates, VBAC saw an increase in popularity between 1989 and 1993 [20]. However, VBAC also correlates with risks of significant complications. Those complications and associated malpractice lawsuits have led to a decline in VBAC despite the fact that 60–80% of women who attempt a trial of labor after CS have a successful VBAC [21].

Robson groups 6–10 are typically less favorable for vaginal delivery due to increased risks. High CS rates in groups 6 and 7 (containing women with nulliparous and multiparous breech births) are similar to what is reported internationally [22] as these are less favorable for vaginal delivery; however, it is interesting that Blacks have lower CS rates for these groups compared with Whites, Asians, and women with more than one race, which could indicate lack of appropriate care. However, there is an increasing movement to change practice in breech deliveries with increasing support for vaginal delivery [23], so the lower CS rates for Black mothers could be an indicator of this change in practice.

Studies investigating the relationship between race and CS rates internationally have focused on immigrants and provided somewhat conflicting results. Research from Norway [24] and London [25] suggests that groups from Sri Lanka/India, Somalia/Eritrea/Ethiopia, the Philippines, and African, West Indian, Bangladeshi, and Pakistani women were all at elevated risk of CS. However, research from Spain suggests that Maghrebian immigrants have a lower risk of CS [26]. These international studies also suggest that type of hospital and difficulties handling the deliveries as well as maternal request for CS may confound observed differences.

### Clinical Implications

Additional research should be conducted into underlying causes of these differences before results can be confidently used in clinical practice. However, clinicians should make efforts to reduce primary CS among low-risk mothers, particularly Black and Asian mothers. Furthermore, among populations that are less favorable for delivery such as breech deliveries, care should be given to ensure Black mothers are receiving optimal care. Finally, despite health care providers’ best intentions, implicit bias may affect care [27]. Providers should consider implicit bias assessment and training to identify any unconscious bias and address it.

### Research Implications

The current study only provides an initial description of the nationwide differences in CS rates by race via the TGCS. Birth certificate data may contain inaccuracies. Future research should collect data prospectively with indications verified by the attending physician to confirm these results. Future research should also examine if there are physiologic, socioeconomic, or cultural differences that may underlie and further explain these results. A widespread adoption of the TGCS in the US would facilitate improved understanding of the quality of labor and delivery. Additionally, a further investigation of factors influencing the higher VBAC rates among NHOPI mothers and if these factors could be used to reduce repeat CS in other populations should be conducted. Finally, the TGCS has been used internationally to also examine maternal and perinatal outcomes [e.g., 28, 29]. Future research should use the TCGS to examine if maternal and perinatal outcomes other than CS are affected by race in a US population.

### Strengths and Limitations

One important limitation to the current study is the limited variable of race. For the current study, the variable of race was limited to 6 categories but each of these groups contains much diversity. Future research should provide a deeper investigation of CS rates in these groups, for example, using variables that identify country/ethnicity of origin or contain more detail when more than one race are reported. Race can also act as a proxy for other variables such as socioeconomic status (SES) that may also affect patterns of care. Future research should explore using CS rates by mother’s SES. Additionally, there are numerous potential confounding factors that may impact the relationship between race and CS such as provider attitudes, type of health care facility, or hospital volume, which are unable to be determined from birth certificate data. Future research should include these in the analysis to further elucidate the underlying causes of racial difference in CS rates. Furthermore, while this is a population-based sample that describes the national trends seen in obstetric practice in the US, it may not reflect practices or trends at an individual institution level.

Strengths of using birth certificate data to compare CS rates include the comprehensive population-based data that comprises all births in the US for a given year. Furthermore, the demographic and medical data included in the current study (such as maternal age, parity, and method of delivery) are considered to be high-quality, carefully recorded data [30]. Finally, this study is one of the first to present a national sample of the US CS data through the lens of the Robson TGCS, a classification system recommended by the WHO and widely used internationally for comparing and monitoring CS rates.

## Conclusions

The results from this study support prior research suggesting there are differences between racial groups in CS rates. Black and Asian mothers have higher rates of CS in lower risk pregnancy groups, suggesting that doctors should work to prevent primary CS in these populations by avoiding medically unnecessary CS and improving implicit bias training for providers. This would be particularly beneficial to overall CS rates as VBAC is still a less popular option; therefore, lowering primary CS may subsequently decrease the number of repeat CS. Furthermore, NHOPI mothers have significantly higher VBAC rates than other groups and research should explore if the factors that lead to increasing VBAC could be applied to other populations to reduce unnecessary CS. Overall, the Robson TGCS is an easy to use, freely available tool that allows for the standardized comparisons of CS rates. While the present study provides an initial description of the nationwide differences in CS rates by race via the Robson TGCS, a more widespread adoption of the system in the US would improve understanding of the quality of labor and delivery in the US.

### Availability of Data and Material

The data is publicly available from the National Vital Statistics System.
